# Interventions to reduce the prescription of inappropriate medicines in older patients

**DOI:** 10.11606/S1518-8787.2019053000781

**Published:** 2019-01-18

**Authors:** Nathalia Serafim dos Santos, Lívia Luize Marengo, Fabio da Silva Moraes, Silvio Barberato

**Affiliations:** I Universidade de Sorocaba. Programa de Pós-Graduação em Ciências Farmacêuticas. Sorocaba, SP, Brasil

**Keywords:** Aged. Health of the Elderly, Patient Medication Knowledge, Inappropriate Prescribing, prevention & control, Review, Idoso, Saúde do Idoso, Conhecimento do Paciente sobre a Medicação, Prescrição Inadequada, prevenção & controle, Revisão

## Abstract

**OBJECTIVE:**

Identify and critically evaluate systematic reviews addressing the effectiveness of interventions to reduce the number of prescriptions of potentially inappropriate medication to older patients.

**METHODS:**

This is an overview of systematic reviews. The studies were searched and selected from Medline, Cochrane Library, Embase, CINAHL, Virtual Health Library, and Web of Science databases, combining the terms *aged, prescriptions, inappropriate prescribing* and *potentially inappropriate medication list* with their entry terms and other related descriptors, published by June 2017. This study included systematic reviews with or without meta-analysis that addressed the effectiveness of any intervention or combined interventions to reduce the number of prescriptions of potentially inappropriate medications to older patients, without restriction in terms of design, language or date of publication of primary studies. AMSTAR – A MeaSurement Tool to Assess systematic Reviews – was used to evaluate the methodological quality of selected systematic reviews. Study selection and the methodological quality evaluation were performed by two independent evaluators, who resolved any divergence by consensus. The main findings were grouped into thematic categories, defined after a content analysis and discussed qualitatively as narrative synthesis.

**RESULTS:**

This study analyzed 24 systematic reviews. In terms of study design and methodological quality evaluation, most were systematic reviews of randomized controlled clinical trials and studies of moderate quality, respectively. The interventions were analyzed in five thematic categories: medication review services, pharmaceutical interventions, computerized systems, educational interventions, and others. The interventions analyzed showed good results and most of them helped reduce the number of prescriptions of potentially inappropriate medication to older patients.

**CONCLUSIONS:**

The systematic reviews included in this overview showed potential benefits of different interventions. However, it was not possible to determine the most effective intervention. Combined interventions are likely to provide better results than isolated interventions.

## RESUMO

**OBJETIVO:**

Identificar e avaliar criticamente revisões sistemáticas sobre a efetividade de intervenções para reduzir a prescrição de medicamentos potencialmente inapropriados para pacientes idosos.

**MÉTODOS:**

*Overview* de revisões sistemáticas. A busca e a seleção dos estudos foram feitas nas bases de dados *Medline*, Biblioteca *Cochrane, Embase, CINAHL*, Biblioteca Virtual em Saúde e *Web of Science*, combinando os termos *aged, prescriptions, inappropriate prescribing* e *potentially inappropriate medication list* com seus sinônimos remissivos e outros descritores associados até junho de 2017. Foram incluídas revisões sistemáticas com ou sem metanálise, que tenham abordado a efetividade de qualquer intervenção ou a combinação de intervenções para reduzir a prescrição de medicamentos potencialmente inapropriados para pacientes idosos, sem restrição quanto ao desenho dos estudos primários, idioma ou data de publicação. Para avaliação da qualidade metodológica das revisões sistemáticas selecionadas, foi utilizado o instrumento *A MeaSurement Tool to Assess systematic Reviews*. A seleção e a avaliação da qualidade metodológica foram realizadas por dois avaliadores independentes. As divergências foram superadas por consenso. Os principais achados foram agrupados em categorias temáticas, definidas com base em análise de conteúdo e discutidas qualitativamente na forma de síntese narrativa.

**RESULTADOS:**

Vinte e quatro revisões sistemáticas foram incluídas no estudo. Quanto ao desenho do estudo e à avaliação da qualidade metodológica, prevaleceram revisões sistemáticas de ensaios clínicos controlados randomizados e estudos de qualidade moderada, respectivamente. As intervenções foram analisadas em cinco categorias temáticas: serviços de revisão de medicamentos, intervenções farmacêuticas, sistemas informatizados, intervenções educacionais e outras. As intervenções estudadas apresentaram bons resultados e a maioria contribuiu para reduzir a prescrição de medicamentos inapropriados para pacientes idosos.

**CONCLUSÕES:**

As revisões sistemáticas incluídas nesse *overview* apontaram benefícios potenciais de diferentes intervenções. No entanto, não foi possível determinar qual a mais efetiva. É provável que intervenções multifacetadas alcancem resultados melhores do que intervenções isoladas.

## INTRODUCTION

Among current global challenges, one trend is that world population ages rapidly, and this demographic transition will affect almost every aspect of society^1^. According to estimates, the number of people aged 60 and over will increase from 962 million in 2017 to 2.1 billion in 2050 and 3.1 billion in 2100^a^.

This population growth poses significant challenges for health systems, increasing the demand for health resources, including medication^2^. The greater the number of items used by a patient, the greater the chances of such patient being submitted to therapy with potentially inappropriate medication^3^.

Prescription of potentially inappropriate medication occurs when the risk of adverse events outweighs the clinical benefit. It also refers to overuse, prescription of multiple drugs with known interactions, incorrect indication or dose, and drug taken longer than necessary^4^ ^,^ ^5^.

Adverse events and drug interactions cause significant morbidity and mortality, especially in older patients as they present alterations in body composition and renal and hepatic functions^6^ ^-^ ^8^.

Prescription of potentially inappropriate medication to older patients has received special attention from health professionals, care providers, researchers, and health policymakers worldwide^9^. Therefore, this study aimed to identify and critically evaluate systematic reviews addressing the effectiveness of interventions to reduce prescriptions of potentially inappropriate medication to older patients.

## METHODS

### Study Design

This is an overview of systematic reviews addressing the effectiveness of interventions to reduce the number of prescriptions of potentially inappropriate medication to older patients.

### Eligibility

The inclusion criteria of this overview were:

- Participants: older patients (≥65 years) who have received drug prescription.
- Interventions: the ones described in the selected studies aiming to reduce the number of prescriptions of potentially inappropriate medication to elderly patients.
- Comparators: usual care to elderly patients or comparison to different interventions.
- Outcomes: primary and secondary outcomes evaluated in the systematic reviews included in this study.
- Study types: systematic reviews with or without meta-analysis that addressed the effectiveness of any intervention or combination of interventions to reduce the number of prescriptions of potentially inappropriate drugs to elderly patients, without restriction in terms of design of primary studies.

### Exclusion Criteria

This overview excluded reviews based on the following criteria: a) abstracts for conference papers and protocols of systematic reviews; b) reviews exclusively based on gray literature; c) studies focused on a specific clinical condition or related to a particular medication or therapeutic class; d) systematic reviews addressing exclusively under-use of medications or interventions to improve treatment adherence; e) systematic reviews that have been updated, without loss of relevant information.

### Search Method for Study Identification

The studies were searched and selected from Medline, Cochrane Library, Embase, CINAHL, Virtual Health Library, and Web of Science databases, combining the terms *aged, prescriptions, inappropriate prescribing* and *potentially inappropriate medication list* with their entry terms and other related descriptors without restriction in terms of study language or date of publication published by June 2017. Box 1 shows the full list of descriptors and Box 2 shows the search strategy in Medline database.

**Box 1:** Descriptors used in database search.

MeSH terms			
Aged	Prescriptions	Inappropriate prescribing	Potentially inappropriate medication list
Entry terms			
Elderly	Prescription Prescriptions, non-drug Non-drug prescription Non-drug prescriptions Prescription, non-drug Prescriptions, non drug Prescriptions, nondrug Nondrug prescription Nondrug prescriptions Prescription, nondrug	Inappropriate prescribings Prescribing, inappropriate Prescribings, inappropriate Inappropriate prescriptions Inappropriate prescription Prescription, inappropriate Prescriptions, inappropriate Over prescribing Over prescribings Prescribing, over Prescribings, over	PIM List PIM Lists Potentially inappropriate medications Inappropriate medication, potentially Inappropriate medications, potentially Medication, potentially inappropriate Medications, potentially inappropriate Potentially inappropriate medication Beers criteria Beers potentially inappropriate medications De Beers criteria Beers criteria, de STOPP (Screening Tool of Older Person's Potentially Inappropriate Prescriptions) STOPP (Screening Tool of Older Person's Potentially Inappropriate Prescriptions) Screening Tool of Older Person's Potentially Inappropriate Prescriptions STOPP STOPP START Criteria Criteria, STOPP START Criterias, STOPP START START Criteria, STOPP START Criterias, STOPP STOPP START Criterias Medication appropriateness index Appropriateness index, medication Appropriateness indices, medication Index, medication appropriateness Indices, medication appropriateness Medication appropriateness indices

**Box 2:** Search strategy in Medline database (via PubMed).

Identification	Search terms
#1	Elderly OR aged OR frail elderly
#2	Prescriptions OR prescription OR prescriptions, non-drug OR non-drug prescription OR non-drug prescriptions OR prescription, non-drug OR prescriptions, non drug OR prescriptions, nondrug OR nondrug prescription OR nondrug prescriptions OR prescription, nondrug OR drug prescriptions OR drug prescription OR drug prescribing OR prescribing, drug OR prescribing OR prescri*
#3	Inappropriate Prescribing OR Inappropriate Prescribings OR Prescribing, Inappropriate OR Prescribings, Inappropriate OR Inappropriate Prescriptions OR Inappropriate Prescription OR Prescription, Inappropriate OR Prescriptions, Inappropriate OR Over Prescribing OR Over Prescribings OR Prescribing, Over OR Prescribings, Over OR Potentially Inappropriate Medication List OR potentially inappropriate medications OR beers OR start OR stopp OR medication appropriateness index OR nurse* OR nursing OR pharmacist* OR pharmaceutical OR intervention* OR clinical decision making
#4	Systematic[sb]
#5	#1 AND #2 AND #3 AND #4

### Study Selection

First, the titles and abstracts of searched reviews were evaluated to identify the studies that met the eligibility criteria. Then, full texts were analyzed and the references were reviewed to identify further relevant studies. Both stages were performed by two independent reviewers, and the divergences were resolved by consensus.

### Data Extraction

Information was extracted about study population, type of intervention, professionals involved in the intervention, comparative treatment, outcome measures, and design of the studies included in the systematic reviews. Complementary information about diseases, sites where the interventions were implemented, and the tools used to assess the prescription of potentially inappropriate medication was also extracted, when available.

Data extraction was performed by the first reviewer and the information obtained was subsequently checked by a second reviewer. The divergences were resolved by consensus.

### Quality Assessment

A MeaSurement Tool to Assess systematic Reviews (AMSTAR) was used for the methodological quality evaluation of the selected systematic reviews^10^. This instrument was specifically designed to evaluate systematic reviews and includes 11 items with four possible answers each. Every question with affirmative answer receives score 1. The systematic reviews selected were assessed by independent reviewers, and the divergences were resolved by consensus.

Based on the consensus score, the systematic reviews were classified as three levels: low methodological quality (score 0 to 3), moderate methodological quality (score 4 to 7), and high methodological quality (score 8 to 11)^11^.

### Data Analysis

The main results of the systematic reviews were grouped into thematic categories and discussed qualitatively as narrative synthesis. The method of content analysis was adopted to define the thematic categories^12^ ^,^ ^13^.

Extracted data were based on the results from each systematic review. Studies of multiple approaches were discussed under more than one thematic category. Discrepancies in the classification of interventions were resolved by consensus. The interventions identified and their results were described in narrative. Detailed information was extracted and systematized to discuss possible discrepant results of the interventions.

No meta-analyses or other quantitative analyses were performed because of the heterogeneity of the studies, considering their different designs, types of intervention, outcomes and measures.

## RESULTS

### Study Selection

In total, 1,850 studies were identified in the databases, and 302 duplicates were removed, resulting in 1,548 studies submitted to title and abstract screening. This initial screening removed 1,487 studies that did not meet the selection criteria. Later, after fully reading 61 eligible studies, 37 were excluded because they did not meet the inclusion criteria, resulting in 24 studies selected for this review. The flow diagram in Figure shows the study selection process.

**Figure:**
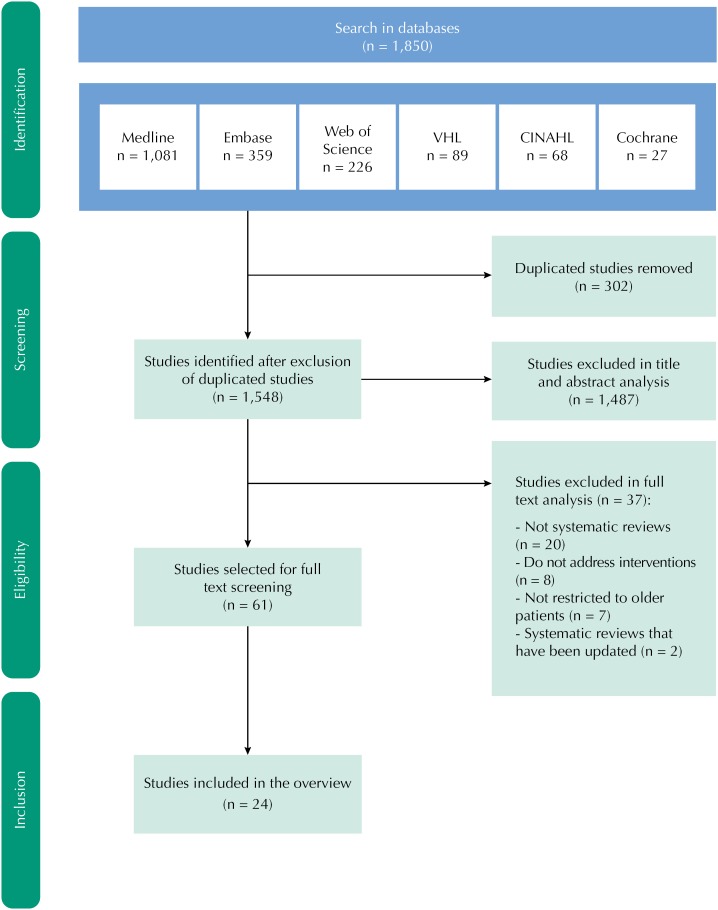
Flow diagram showing the selection process of systematic reviews about interventions to reduce the number of prescriptions of potentially inappropriate medications to older patients.

Two systematic reviews from Cochrane^14^ ^,^ ^15^ retrieved in the search were subsequently updated. This overview included the two most recent studies^16^ ^,^ ^17^ only.

### Characteristics of Studies

Table 1 shows the main characteristics of the studies. In terms of design of primary studies included in the systematic reviews, most of them were randomized controlled clinical trials. The number of primary studies included in the systematic reviews varied from four to 116. These studies were conducted in different settings, including: primary care, community, hospitals, nursing homes, and long-term care facilities. Fifteen systematic reviews were published in 2014–2017.

**Table 1:** Characteristics of studies included in this overview.

Author (year)	Study design	Number of studies included	Interventions	Setting	AMSTAR score
Alldred et al.^16^ (2016)	Randomized controlled clinical trial	12	Educational Interventions Medication review Computerized systems	Long-term care unit	8/11
Castelino et al.^19^ (2009)	Randomized controlled clinical trial	12	Pharmaceutical intervention Medication review	Hospital	5/11
Cooper et al.^2^ (2015)	Randomized controlled clinical trial	12	Pharmaceutical intervention Medication review	Hospital	9/11
Forsetlund et al.^5^ (2011)	Randomized controlled clinical trial	20	Educational Intervention Medication review	Nursing home	7/11
Gutierrez Valencia et al.^7^ (2016)	Prospective studies (not necessarily controlled and randomized) with interventions	18	Pharmaceutical interventions Educational Interventions Computerized systems Medication review	Hospital	7/11
Iankowitz et al.^46^ (2015)	Randomized controlled clinical trial, quasi-experimental study	5	Computerized systems	Hospital	9/11
Johansson et al.^27^ (2016)	Randomized controlled clinical trial, randomized controlled trial, non-randomized controlled clinical trial, cohort study, case-control study	25	Pharmaceutical interventions Medication review	Hospital	3/11
Kaur et al.^20^ (2009)	Randomized controlled clinical trial, prospective study, cohort study, survey, retrospective and prospective case series	24	Pharmaceutical interventions Educational Interventions Computerized systems Medication review Other interventions	Hospital	6/11
Loganathan et al.^28^ (2011)	Randomized controlled clinical trial and non-randomized controlled clinical trial	16	Pharmaceutical interventions Educational Interventions Computerized systems Medication review	Nursing home	8/11
Loh et al.^29^ (2016)	Randomized controlled clinical trial	25	Medication review	Community	3/11
Maeda^24^ (2009)	Randomized controlled trials, controlled clinical trials	28	Medication review	Community Hospital Nursing home	5/11
Marasinghe^45^ (2015)	Randomized controlled clinical trial, cohort studies, retrospective and prospective studies	7	Computerized systems	Long-term care unit	6/11
Michelazzo et al.^21^ (2017)	Case series	19	Pharmaceutical intervention Educational Intervention Medication review	Hospital Community Nursing home	3/11
Olaniyan et al.^47^ (2015)	Retrospective non-randomized study, Retrospective randomized study	68	Pharmaceutical intervention Computerized systems	Primary care	9/11
Page et al.^52^ (2016)	Randomized controlled clinical trial	116	Other interventions	Hospital	10/11
Patterson et al.^17^ (2014)	Randomized controlled clinical trial, non-randomized controlled clinical trials, controlled before-after studies, interrupted time series	10	Pharmaceutical interventions Computerized systems	Hospital	7/11
Riordan et al.^22^ (2016)	Randomized controlled clinical trial, quasi-randomized controlled clinical trial, controlled before-after studies, interrupted time series	5	Pharmaceutical interventions Medication review	Primary care	3/11
Rollason e Vogt^25^ (2003)	Randomized controlled clinical trial	14	Pharmaceutical interventions Medication review	Hospital	3/11
Shade et al.^48^ (2014)	Randomized controlled clinical trial and non-randomized clinical trial	67	Computerized systems	Community	7/11
Thiruchelvam et al.^23^ (2017)	Randomized controlled clinical trial and observational studies	22	Medication review	Hospital	5/11
Tjia et al.^33^ (2013)	Randomized controlled clinical trial and non-randomized clinical trial, before-after studies, case series	36	Medication review	Hospital Nursing home	3/11
Verrue et al.^30^ (2009)	Controlled trials (randomized or non-randomized)	8	Pharmaceutical interventions Medication review	Nursing home	3/11
Walsh et al.^35^ (2016)	Controlled trials (randomized or non-randomized)	4	Pharmaceutical interventions Medication review	Hospital	7/11
Yourman et al.^50^ (2008)	Randomized controlled clinical trial	10	Computerized systems	Hospital	3/11

AMSTAR: A MeaSurement Tool to Assess systematic Reviews

### Methodological Quality Assessment

The methodological quality assessment according to AMSTAR found most systematic reviews of moderate quality (n = 10). Six studies presented enough score to be considered of high methodological quality and eight were classified as low quality studies. The last column of Table 1 shows the scores attributed to each systematic review.

### Synthesis of Interventions Grouped into Thematic Categories

The interventions identified in selected systematic reviews were grouped into five thematic categories: medication review services (n = 16), pharmaceutical interventions (n = 10), computerized systems (n = 10), educational interventions (n = 8), and other interventions (n = 2).

Medication review services were not analyzed in the category of pharmaceutical interventions because, although they may be conducted by pharmacists, they often include other health professionals.

Table 2 shows the thematic categories addressed in each systematic review included in this study.

**Table 2:** Thematic categories addressed in the systematic reviews about interventions to reduce the number of prescriptions of inappropriate medications to older patients evaluated in this overview.

Author (year)	Interventions				
	Medication review services	Pharmaceutical interventions	Computerized systems	Educational interventions	Other interventions
Alldred et al.^16^ (2016)	X		X	X	
Castelino et al.^19^ (2009)	X	X			
Cooper et al.^2^ (2015)	X	X			
Forsetlund et al.^5^ (2011)	X			X	
Gutierrez Valencia et al.^7^ (2016)	X		X	X	
Iankowitz et al.^46^ (2015)			X		
Johansson et al.^27^ (2016)	X				
Kaur et al.^20^ (2009)	X	X	X	X	X
Loganathan et al.^28^ (2011)	X	X	X	X	
Loh et al.^29^ (2016)	X				
Maeda^24^ (2009)	X				
Marasinghe^45^ (2015)			X		
Michelazzo et al.^21^ (2017)	X	X		X	
Olaniyan et al.^47^ (2015)			X		
Page et al.^52^ (2016)				X	X
Patterson et al.^17^ (2014)		X	X		
Riordan et al.^22^ (2016)	X	X			
Rollason e Vogt^25^ (2003)	X	X			
Shade et al.^48^ (2014)			X	X	
Thiruchelvam et al.^23^ (2017)	X				
Tjia et al.^33^ (2013)	X				
Verrue et al.^30^ (2009)	X	X			
Walsh et al.^35^ (2016)		X			
Yourman et al.^50^ (2008)			X		

### Medication Review Services

Medication review includes many interventions that can be performed by prescribers (self-review) or other health professionals (usually physicians, pharmacists, and nurses), alone or combined with others, that provide prescribers with recommendations to improve the quality of prescription and increase drug use safety^18^.

Although the descriptions of medication reviews varied in the studies analyzed, the process generally involved a systematic assessment of the patient's pharmacotherapeutic needs and prescribed drugs, followed by recommendations to optimize the dosage. Promising results were observed in interventions involving pharmacists, with the authors emphasizing the importance of training these professionals on tools to identify inappropriate medications for older patients^19^.

Medication review with a clinical pharmacist may have a positive influence on the use of medicines. These interventions, either alone or combined with others, can reduce the use of potentially inappropriate medications by older patients in different settings^2^ ^,^ ^5^ ^,^ ^7^ ^,^ ^20^ ^-^ ^25^.

On the other hand, Holland et al.^26^ evaluated the impact of medication review on hospital admissions and mortality and found no positive effect.

The studies used different methods for medication review, whose methodology is a key issue in interventions, and it is not clear which would be the most appropriate^27^. The selection of outcomes to be measured in primary studies has also influenced the results^28^.

Many studies do not mention whether or not the recommended changes in prescription after the medication review were accepted by the prescriber. According to the authors, this is a critical parameter in medication review evaluation, since it describes actual changes in patient treatment as a result of the intervention^29^ ^,^ ^30^. These rates varied from 39.0%^31^ to 91.6%^32^, with possible low acceptance justified by the indirect contact of the pharmacist with the general practitioner, demonstrating the importance of communication in the multidisciplinary health team. The heterogeneity in study design and the quality of studies are obstacles to conclude whether medication reviews by pharmacists are more effective than interdisciplinary interventions^33^.

### Pharmaceutical Intervention

It refers to the clinical practice of pharmacists, often integrated with physicians, nurses and other members of the health team, to solve or prevent problems that interfere or may interfere in the pharmacotherapy, which is part of the care process. The main objective of this activity is the prevention of errors in drug prescription, dispensing and administration, with a critical role in promoting the rational use of medication by ensuring proper pharmacotherapy with safe therapeutic results and minimizing unfavorable outcomes^34^.

Pharmaceutical care seems to improve prescriptions to older patients taking different medications at the same time (polypharmacy), especially when a multidisciplinary element is included in care^17^. The practice of pharmacists has been associated with benefits in different contexts, including primary care^22^, hospitals^28^ ^,^ ^35^, and nursing homes^28^ ^,^ ^30^. However, the role of a pharmacist in a multidisciplinary team needs to be more valued to help achieve the expected results^22^.

Castelino et al.^19^ highlighted the importance of training pharmacists on validated tools to identify inappropriate medications. They also argued that the quality of a prescription can improve when these professionals assume a more active role in this process, since intervention studies generally focus on identifying failures after prescription.

On the other hand, Cooper et al.^2^ found no evidence of benefit from pharmaceutical interventions on adverse events and admissions. Inadequate selection of outcome measures may have influenced the evaluation of efficacy of such interventions, whose therapeutic adequacy has been analyzed more often than other relevant health outcomes^21^ ^,^ ^28^.

### Computerized Systems

Computerized systems allow electronic prescription and records about the medications taken by every patient; besides, they issue risk alerts and provide information about drug interactions. These systems are often used at two different levels: when making decisions and issuing alerts to pharmacies when dispensing drugs^20^ ^,^ ^36^ ^-^ ^44^.

Information and communications technologies are increasingly used to optimize prescriptions in different settings^16^. Most studies have demonstrated the effectiveness of computerized systems^7^ ^,^ ^20^ ^,^ ^28^ ^,^ ^45^, including meta-analysis^46^.

Collaborative implementation of computerized systems and other interventions can optimize the safety of medication use in primary care and improve health outcomes^47^. Patterson et al.^17^ also highlighted a study^36^ whose results were positive and showed that most pharmaceutical interventions involved a multidisciplinary component and interventions through computerized systems.

Although studies indicate a significant reduction of potentially inappropriate drug prescriptions, computerized systems may not provide a full picture of medication use by older patients, since other drugs may be purchased at pharmacies not participating in the intervention or as over-the-counter medicines^48^. Gurwitz et al.^49^ also pointed out that the high number of alerts in a system can cause prescribers to ignore them, with a negative impact on the prescriptions.

Successful interventions with computerized systems should be tested and improved in different settings to enhance patient safety and minimize adverse effects. Regular medication review and timely interventions in prescriptions are essential in clinical practice to address the increasing challenges involving prescriptions to older patients^50^.

### Educational Interventions

Educational interventions can be conducted in different ways, including educational sessions for health professionals aiming to reduce drug use; distribution of educational materials; training to expand the knowledge and skills of patients, caregivers, and health professionals; educational programs for prescribers or consumers; and patient education to optimize polypharmacy^17^ ^,^ ^51^ ^,^ ^52^.

Educational interventions may reduce inappropriate drug prescription^5^ ^,^ ^20^ ^,^ ^48^ and hospitalization period^53^, either alone or combined with other interventions^7^.

Loganathan et al.^28^ analyzed six studies^54^ ^-^ ^59^ that adopted strategies of educational intervention, resulting in improvements in prescriptions. These interventions included face-to-face academic detailing, interaction between the prescriber and a group of specialists, workshops for nurses, and family education.

However, educational interventions have been studied more in terms of changes in therapy than for other outcomes related to the quality of life of patients, costs and use of health services^21^.

### Other Interventions

Two systematic reviews addressed other interventions to reduce the number of prescriptions of potentially inappropriate medications to older patients, including: geriatric medicine services^20^, regulatory interventions^20^, and deprescription^52^.

In a study conducted by Kaur et al.^20^, all interventions involving geriatric medicine services resulted in improvements for patients. The authors also highlighted two studies on regulatory interventions that reduced the number of potentially inappropriate drug prescription: one in which pharmacy service provision became mandatory in nursing homes in Canada^60^, and one that assessed the impact of restrictive measures adopted in the Australian form Pharmaceutical Benefits Scheme (PBS), which lists prescription drugs subsidized by the government^61^.

Page et al.^52^ presented data about deprescription interventions aiming to reduce polypharmacy and extend longevity. Although the authors state further studies are required, their findings suggest that individualized interventions help reduce inappropriate polypharmacy and seem to be safe and feasible.

## DISCUSSION

### Main Findings

This overview of systematic reviews summarizes evidence of interventions to reduce the number of prescriptions of potentially inappropriate medications to older patients, identifying knowledge gaps and providing insight for policy making and future studies.

Medication review prevailed among the types of intervention in this overview. Most studies support the benefits of this intervention, especially when using validated tools. It has produced better results when associated with other interventions^2^ ^,^ ^5^ ^,^ ^16^ ^,^ ^20^ ^,^ ^22^. On the other hand, choice of outcome measures^28^, study design^33^, and methodological quality^33^ have often been obstacles when assessing the efficacy of this intervention.

The practice of pharmacists to reduce the number of prescriptions of potentially inappropriate medications to older patients is also highlighted in the literature. In this type of intervention, pharmacists can act with autonomy to change the prescription, or act passively, identifying problems related to medications and recommending changes to the prescriber, who makes the final decision^62^. The practice of pharmacists seems to improve prescription in different settings (hospitals, primary care, and nursing homes), particularly when inserted in multidisciplinary teams.

The use of computerized systems presented the best evidence of benefit in selected studies. These resources have been increasingly used in different scenarios, supporting either clinical decision making or the pharmacotherapeutic analysis in drug dispensing^16^ ^,^ ^20^ ^,^ ^45^ ^,^ ^46^ ^,^ ^50^.

Educational interventions can be designed for prescribers, other health professionals, patients or caregivers. Whether alone or combined with other interventions, they have been effective in reducing inappropriate use of medications^5^ ^,^ ^20^ ^,^ ^28^.

Regulatory policies that have produced positive results include the potential benefits of eliminating subsidies from potentially inappropriate medications to influence prescribing^61^ and making pharmacy services mandatory in nursing homes in Canada^60^.

Geriatric medicine services^20^ and deprescription^52^ have also resulted in improvements for patients.

### Strengths and Limitations

One of the reasons for an overview of systematic reviews was to identify different interventions already implemented and evaluated in clinical practice and check which ones present the best evidence of benefit to promote the rational use of medications among older patients.

The strengths of this study include: description of interventions to reduce the number of prescriptions of potentially inappropriate medications to older patients, based on the evidence available; comprehensive search structured according to the PICOS (patient, intervention, comparison, outcomes and study type) method; methodological quality assessment of the studies; and no restrictions regarding language or date of publication.

The quality of systematic reviews, predominantly moderate, must be confirmed by further studies designed with more methodological rigor. It means that although each type of intervention reported relevant results, it was not possible to reach definitive conclusions about the most effective intervention to reduce the number of prescriptions of potentially inappropriate medications to older patients.

In addition, overviews of systematic reviews are subject to important limitations, especially when addressing complex issues and heterogeneous outcomes. When systematizing the results of almost 600 primary studies, particularities of individual studies may have been lost or neglected by the authors of the respective systematic reviews.

### Implications for Practice

Evidence supports that the use of computerized systems reduces the prescription and dispensing of inappropriate drugs to older patients. Medication review, either by health professionals alone or in a multidisciplinary team, has presented promising results. However, the acceptance of recommendations by prescribers plays a critical role in the achievement of results, so there is no consensus on which is the best methodology. Interventions conducted by pharmacists may also improve drug prescription to older patients. It stresses the trend of pharmaceutical care implementation and values the clinical role of pharmacists integrated with other health professionals.

A combination of interventions was supported by the evidence of educational interventions^5^ ^,^ ^7^ and in the evaluation of the effectiveness of computerized systems^47^ ^,^ ^50^ and medication review services^2^ ^,^ ^5^ ^,^ ^16^ ^,^ ^20^ ^,^ ^22^.

Ideally, interventions should have been evaluated using clinically relevant outcomes, such as mortality, quality of life, or utilization of health services. But these outcomes were not evaluated in most primary studies included in the systematic reviews. Then, the interventions described can improve the prescription and enhance safety in the use of medications, but cannot confirm the clinical benefits achieved.

### Implications for Research and Health Policies

A detailed description of the interventions, the settings where they were studied, the implementation strategies, and the results achieved is critical to reinforce the evidence and support the selection and implementation of the best interventions and their reproduction in different contexts^2^ ^,^ ^17^. Also important, the cost of interventions should be compared to the economic impact of potentially inappropriate drug prescriptions to sensitize managers and policy makers.

Patient preferences, beliefs and behaviors may also be considered, as well as economic assessments and other aspects of health policies. Qualitative studies involving health professionals and patients can provide important information about obstacles for the implementation or acceptance of an intervention^27^. Interviews with prescribers can help understand their reasons for not accepting recommendations and alerts from computerized systems that support drug prescription.

Instead of evaluating the reduction in the number of potentially inappropriate drug prescriptions, a trend is observed towards the assessment of whether polypharmacy can be considered appropriate (when drugs were prescribed and used according to the best evidence) or inappropriate (when inappropriately prescribed or the intended benefits have not been achieved)^17^.

Future studies should ensure greater methodological rigor in the evaluation of interventions to reduce the number of potentially inappropriate drug prescription to older patients. Further studies are required which should investigate the effectiveness of individual and combined interventions. Studies comparing different interventions can also establish the real value of each intervention.

## CONCLUSIONS

The systematic reviews included in this overview showed potential benefits from different interventions in reducing the number of prescriptions of potentially inappropriate medications to older patients. The results expected from each intervention were discussed in this overview, and although it was not possible to determine which one is the most effective, combined interventions are likely to achieve better results than isolated interventions.

Knowledge gaps reveal relevant topics for future studies to be conducted with the higher methodological rigor.

In order to increase the safety of medication use by older patients, organizational and structural measures can be planned and implemented in health services, such as: computerized systems to support drug prescription and dispensing, training on the use of validated tools for the detection of potentially inappropriate drugs, procedures and explicit routines for medication review, continuing education for health professionals, and geriatric medicine services.

It should be noted that the deployment of any intervention can become a reality with the involvement of all stakeholders: policy makers, administrators, health professionals, patients, and caregivers.

## Footnotes

a United Nations Department of Economic and Social Affairs. World population prospects: the 2017 revision. New York; 2017[cited 2017 Dec 21]. Available from: https://www.un.org/development/desa/publications/world-population-prospects-the-2017-revision.htm

## References

[1] Beard JR, Carvalho IA, Sumi Y, Officer A, Thiyagarajan JA (2017). Healthy ageing: moving forward. Bull World Health Organ.

[2] Cooper JA, Cadogan CA, Patterson SM, Kerse N, Bradley MC, Ryan C (2015). Interventions to improve the appropriate use of polypharmacy in older people: a Cochrane systematic review. BMJ Open.

[3] Santos APAL, Silva DT, Alves-Conceição V, Antoniolli AR, Lyra DP (2015). Conceptualizing and measuring potentially inappropriate drug therapy. J Clin Pharm Ther.

[4] Fond G, Fajula C, Dassa D, Brunel L, Lancon C, Boyer L (2016). Potentially inappropriate psychotropic prescription at discharge is associated with lower functioning in the elderly psychiatric inpatients: a cross-sectional study. Psychopharmacology (Berl).

[5] Forsetlund L, Eike MC, Gjerberg E, Vist GE (2011). Effect of interventions to reduce potentially inappropriate use of drugs in nursing homes: a systematic review of randomised controlled trials. BMC Geriatr.

[6] Clyne B, Bradley MC, Hughes CM, Clear D, McDonnell R, Williams D (2013). Addressing potentially inappropriate prescribing in older patients: development and pilot study of an intervention in primary care (the OPTI-SCRIPT study). BMC Health Serv Res.

[7] Gutierrez Valencia M, Martinez Velilla N, Lacalle Fabo E, Beobide Telleria I, Larrayoz Sola B, Tosato M (2016). Intervenciones para optimizar el tratamiento farmacológico en ancianos hospitalizados: una revisión sistemática. Rev Clin Esp.

[8] Tommelein E, Petrovic M, Somers A, Mehuys E, Cammen T, Boussery K (2016). Older patients’ prescriptions screening in the community pharmacy: development of the Ghent Older People's Prescriptions community Pharmacy Screening (GheOP^3^S) tool. J Public Health (Oxf).

[9] Soares MA, Fernandez-Llimos F, Cabrita J, Morais J (2011). Critérios de avaliação de prescrição de medicamentos potencialmente inapropriados: uma revisão sistemática. Acta Med Port..

[10] Shea BJ, Grimshaw JM, Wells GA, Boers M, Andersson N, Hamel C (2007). Development of AMSTAR: a measurement tool to assess the methodological quality of systematic reviews. BMC Med Res Methodol.

[11] Biondi-Zoccai G (2016). Umbrella reviews: evidence synthesis with overviews of reviews and meta-epidemiologic studies.

[12] Bardin L (2011). Content analysis.

[13] Minayo MCS (2014). O desafio do conhecimento: pesquisa qualitativa em saúde.

[14] Patterson SM, Hughes C, Kerse N, Cardwell CR, Bradley MC (2012). Interventions to improve the appropriate use of polypharmacy for older people. Cochrane Database Syst Rev..

[15] Alldred DP, Raynor DK, Hughes C, Barber N, Chen TF, Spoor P (2013). Interventions to optimise prescribing for older people in care homes. Cochrane Database Syst Rev.

[16] Alldred DP, Kennedy MC, Hughes C, Chen TF, Miller P (2016). Interventions to optimise prescribing for older people in care homes. Cochrane Database Syst Rev.

[17] Patterson SM, Cadogan CA, Kerse N, Cardwell CR, Bradley MC, Ryan C (2014). Interventions to improve the appropriate use of polypharmacy for older people. Cochrane Database Syst Rev..

[18] Blenkinsopp A, Bond C, Raynor DK (2012). Medication reviews..

[19] Castelino RL, Bajorek BV, Chen TF (2009). Targeting suboptimal prescribing in the elderly: a review of the impact of pharmacy services. Ann Pharmacother.

[20] Kaur S, Mitchell G, Vitetta L, Roberts MS (2009). Interventions that can reduce inappropriate prescribing in the elderly: a systematic review. Drugs Aging.

[21] Michelazzo MB, Milovanovic S, Boccia S (2017). A systematic review of case-series studies on the effectiveness of interventions to reduce polypharmacy and its adverse consequences in the elderly. Epidemiol Biostat Public Health.

[22] Riordan DO, Walsh KA, Galvin R, Sinnott C, Kearney PM, Byrne S (2016). The effect of pharmacist-led interventions in optimising prescribing in older adults in primary care: a systematic review. SAGE Open Med.

[23] Thiruchelvam K, Hasan SS, Wong PS, Kairuz T (2017). Residential aged care medication review to improve the quality of medication use: a systematic review. J Am Med Dir Assoc.

[24] Maeda K (2009). Systematic review of the effects of improvement of prescription to reduce the number of medications in the elderly with polypharmacy. Yakugaku Zasshi.

[25] Rollason V, Vogt N (2003). Reduction of polypharmacy in the elderly: a systematic review of the role of the pharmacist. Drugs Aging.

[26] Holland R, Desborough J, Goodyer L, Hall S, Wright D, Loke YK (2008). Does pharmacist-led medication review help to reduce hospital admissions and deaths in older people? A systematic review and meta-analysis. Br J Clin Pharmacol.

[27] Johansson T, Abuzahra ME, Keller S, Mann E, Faller B, Sommerauer C (2016). Impact of strategies to reduce polypharmacy on clinically relevant endpoints: a systematic review and meta-analysis. Br J Clin Pharmacol.

[28] Loganathan M, Singh S, Franklin BD, Bottle A, Majeed A (2011). Interventions to optimise prescribing in care homes: systematic review. Age Ageing.

[29] Loh ZW, Cheen MH, Wee HL (2016). Humanistic and economic outcomes of pharmacist-provided medication review in the community-dwelling elderly: a systematic review and meta-analysis. J Clin Pharm Ther.

[30] Verrue CL, Petrovic M, Mehuys E, Remon JP, Vander Stichele R (2009). Pharmacists’ interventions for optimization of medication use in nursing homes: a systematic review. Drugs Aging.

[31] Roberts MS, Stokes JA, King MA, Lynne TA, Purdie DM, Glasziou PP (2001). Outcomes of a randomized controlled trial of a clinical pharmacy intervention in 52 nursing homes. Br J Clin Pharmacol.

[32] Furniss L, Burns A, Craig SK, Scobie S, Cooke J, Faragher B (2000). Effects of a pharmacist's medication review in nursing homes: randomised controlled trial.

[33] Tjia J, Velten SJ, Parsons C, Valluri S, Briesacher BA (2013). Studies to reduce unnecessary medication use in frail older adults: a systematic review. Drugs Aging.

[34] Ribeiro VF, Sapucaia KCG, Aragão LAO, Bispo ICS, Oliveira VF, Lalves BL (2015). Realização de intervenções farmacêuticas por meio de uma experiência em farmácia clínica. Rev Bras Farm Hosp Serv Saude.

[35] Walsh K, O’Riordan D, Kearney PM, Timmons S, Byrne S (2016). Improving the appropriateness of prescribing in older patients: a systematic review and meta-analysis of pharmacists’ interventions in secondary care. Age Ageing.

[36] Tamblyn R, Huang A, Perreault R, Jacques A, Roy D, Hanley J (2003). The medical office of the 21st century (MOXXI): effectiveness of computerized decision-making support in reducing inappropriate prescribing in primary care. CMAJ.

[37] Devine EB, Hansen RN, Wilson-Norton JL, Lawless NM, Fisk AW, Blough DK (2010). The impact of computerized provider order entry on medication errors in a multispecialty group practice. J Am Med Inform Assoc.

[38] Nemeth LS, Wessell AM (2010). Improving medication safety in primary care using electronic health records. J Patient Saf.

[39] Boockvar KS, Livote EE, Goldstein N, Nebeker JR, Siu A, Fried T (2010). Electronic health records and adverse drug events after patient transfer. Qual Saf Health Care.

[40] Moniz TT, Seger AC, Keohane CA, Seger DL, Bates DW, Rothschild JM (2011). Addition of electronic prescription transmission to computerized prescriber order entry: effect on dispensing errors in community pharmacies. Am J Health Syst Pharm.

[41] Hazlet TK, Lee TA, Hansten PD, Horn JR (2001). Performance of community pharmacy drug interaction software. J Am Pharm Assoc (Wash).

[42] Abramson EL, Bates DW, Jenter C, Volk LA, Barron Y, Quaresimo J (2012). Ambulatory prescribing errors among community-based providers in two states. J Am Med Inform Assoc.

[43] Raebel MA, Charles J, Dugan J, Carroll NM, Korner EJ, Brand DW (2007). Randomized trial to improve prescribing safety in ambulatory elderly patients. J Am Geriatr Soc.

[44] Humphries TL, Nikki C, Chester EA, Magid D, Rocho B (2007). Evaluation of an electronic critical drug interaction program coupled with active pharmacist intervention. Ann Pharmacother.

[45] Marasinghe KM (2015). Computerised clinical decision support systems to improve medication safety in long-term care homes: a systematic review. BMJ Open.

[46] Iankowitz N, Dowden M, Palomino S, Uzokwe H, Worral P (2012). The effectiveness of computer system tools on potentially inappropriate medications ordered at discharge for adults older than 65 years of age: a systematic review. JBI Libr Syst Rev.

[47] Olaniyan JO, Ghaleb M, Dhillon S, Robinson P (2015). Safety of medication use in primary care. Int J Pharm Pract.

[48] Shade MY, Berger AM, Chaperon C (2014). Potentially inappropriate medications in community-dwelling older adults. Res Gerontol Nurs.

[49] Gurwitz JH, Field TS, Rochon P, Judge J, Harrold LR, Bell CM (2008). Effect of computerized provider order entry with clinical decision support on adverse drug events in the long-term care setting. J Am Geriatr Soc.

[50] Yourman L, Concato J, Agostini JV (2008). Use of computer decision support interventions to improve medication prescribing in older adults: a systematic review. Am J Geriatr Pharmacother.

[51] Fulton MM, Allen ER (2005). Polypharmacy in the elderly: a literature review. J Am Acad Nurse Pract.

[52] Page AT, Clifford RM, Potter K, Schwartz D, Etherton-Beer CD (2016). The feasibility and effect of deprescribing in older adults on mortality and health: a systematic review and meta-analysis. Br J Clin Pharmacol.

[53] Pitkälä KH, Juola AL, Kautiainen H, Soini H, Finne-Soveri UH, Bell JS (2014). Education to reduce potentially harmful medication use among residents of assisted living facilities: a randomized controlled trial. J Am Med Dir Assoc.

[54] Fialová D, Topinková E, Gambassi G, Finne-Soveri H, Jónsson PV, Carpenter I (2005). Potentially inappropriate medication use among elderly home care patients in Europe. JAMA.

[55] Beers MH, Ouslander JG, Fingold SF, Morgenstern H, Reuben DB, Rogers W (1992). Inappropriate medication prescribing in skilled-nursing facilities. Ann Intern Med.

[56] Roberts MS, Stokes JA (1998). Prescriptions, practitioners and pharmacists. Med J Aust.

[57] Dartnell JG, Anderson RP, Chohan V, Galbraith KJ, Lyon ME, Nestor PJ (1996). Hospitalisation for adverse events related to drug therapy: incidence, avoidability and costs. Med J Aust.

[58] Goodman M, Lazzarini R (1995). Examination of the feasibility of an ongoing strategy for disposal of unwanted and outdated medicines [abstract].

[59] Blackbourn J (1991). Readmission to Fremantle Hospital: part 2. Drug-related readmissions. Fremantle Hosp Drug Bull.

[60] Lane CJ, Bronskill SE, Sykora K, Dhalla IA, Anderson GM, Mamdani MM (2004). Potentially inappropriate prescribing in Ontario community-dwelling older adults and nursing home residents. J Am Geriatr Soc.

[61] King MA, Roberts MS (2007). The influence of the Pharmaceutical Benefits Scheme (PBS) on inappropriate prescribing in Australian nursing homes. Pharm World Sci.

[62] Meid AD, Lampert A, Burnett A, Seidling HM, Haefeli WE (2015). The impact of pharmaceutical care interventions for medication underuse in older people: a systematic review and meta-analysis. Br J Clin Pharmacol.

